# Supplementary material for: Metagenomic binning with assembly graph embeddings
Source: Bioinformatics. 2022 Aug 16;38(19):4481–7. doi: 10.1093/bioinformatics/btac557 (PMC9525014; doi:10.1093/bioinformatics/btac557)
Supplement: btac557_supplementary_data [file btac557_supplementary_data.zip › amber outputs.zip!/amber outputs/strong100/genome/high_scoring_bins.pdf]

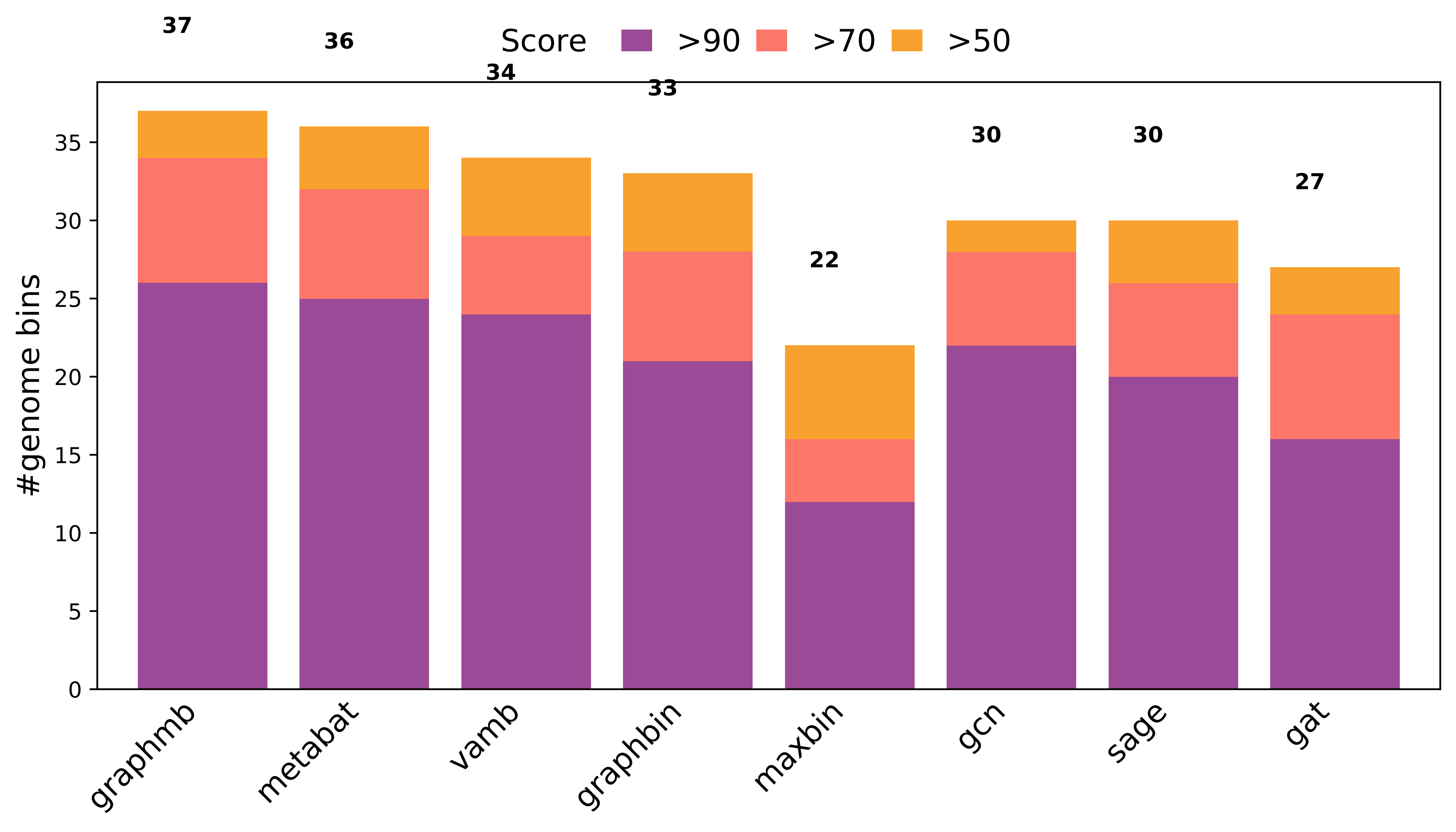

Score
>90
>70
>50
37
36
34
33
22
30
30
27
#genome bins
0
5
10
15
20
25
30
35
graphmb
metabat
vamb
graphbin
maxbin
gcn
sage
gat
